# A Chicken Production Intervention and Additional Nutrition Behavior Change Component Increased Child Growth in Ethiopia: A Cluster-Randomized Trial

**DOI:** 10.1093/jn/nxaa181

**Published:** 2020-07-11

**Authors:** Simone Passarelli, Ramya Ambikapathi, Nilupa S Gunaratna, Isabel Madzorera, Chelsey R Canavan, Abdallah R Noor, Amare Worku, Yemane Berhane, Semira Abdelmenan, Simbarashe Sibanda, Bertha Munthali, Tshilidzi Madzivhandila, Lindiwe M Sibanda, Kumlachew Geremew, Tadelle Dessie, Solomon Abegaz, Getnet Assefa, Christopher Sudfeld, Margaret McConnell, Kirsten Davison, Wafaie Fawzi

**Affiliations:** Department of Nutrition, Harvard TH Chan School of Public Health, Boston, MA, USA; Department of Public Health, Purdue University, West Lafayette, IN, USA; Department of Public Health, Purdue University, West Lafayette, IN, USA; Department of Nutrition, Harvard TH Chan School of Public Health, Boston, MA, USA; Department of Nutrition, Harvard TH Chan School of Public Health, Boston, MA, USA; Department of Nutrition, Harvard TH Chan School of Public Health, Boston, MA, USA; Addis Continental Institute of Public Health, Addis Ababa, Ethiopia; Addis Continental Institute of Public Health, Addis Ababa, Ethiopia; Addis Continental Institute of Public Health, Addis Ababa, Ethiopia; Food, Agriculture and Natural Resources Policy Analysis Network, Pretoria, South Africa; Food, Agriculture and Natural Resources Policy Analysis Network, Pretoria, South Africa; Food, Agriculture and Natural Resources Policy Analysis Network, Pretoria, South Africa; Food, Agriculture and Natural Resources Policy Analysis Network, Pretoria, South Africa; International Livestock Research Institute, Addis Ababa, Ethiopia; International Livestock Research Institute, Addis Ababa, Ethiopia; Ethiopian Institute of Agricultural Research, Addis Ababa, Ethiopia; Ethiopian Institute of Agricultural Research, Addis Ababa, Ethiopia; Department of Global Health and Population, Harvard TH Chan School of Public Health, Boston, MA, USA; Department of Global Health and Population, Harvard TH Chan School of Public Health, Boston, MA, USA; Boston College School of Social Work, Boston, MA, USA; Department of Global Health and Population, Harvard TH Chan School of Public Health, Boston, MA, USA

**Keywords:** child nutrition, agriculture, Ethiopia, chicken, child growth, eggs, diarrhea, anemia, WASH

## Abstract

**Background:**

Chicken production in the context of nutrition-sensitive agriculture may benefit child nutrition in low-income settings.

**Objectives:**

This study evaluated effects of *1*) a chicken production intervention [African Chicken Genetic Gains (ACGG)], and *2*) the ACGG intervention with nutrition-sensitive behavior change communication (BCC) [ACGG + Agriculture to Nutrition (ATONU)], on child nutrition and health outcomes and hypothesized intermediaries.

**Methods:**

Forty ACGG villages received 25 genetically improved chickens and basic husbandry guidance; of these, 20 ACGG + ATONU villages in addition received a nutrition-sensitive behavior change and homegardening intervention; 20 control clusters received no intervention. We assessed effects of the interventions on height-for-age *z* scores (HAZ), weight-for-age *z* scores (WAZ), and weight-for-height *z* scores (WHZ) at 9 (midline) and 18 mo (endline) through unadjusted and adjusted ordinary least squares (OLS) regressions. We examined the interventions’ effects on hypothesized intermediaries including egg production and consumption, dietary diversity, women's empowerment, income, child morbidities, anemia, and chicken management practices through OLS and log binomial models.

**Results:**

Data included 829 children aged 0–36 mo at baseline. ACGG + ATONU children had higher midline HAZ [mean difference (MD): 0.28; 95% CI: 0.02, 0.54] than controls. The ACGG group had higher HAZ (MD: 0.28; 95% CI: 0.05, 0.50) and higher WAZ (MD: 0.18; 95% CI: 0.01, 0.36) at endline than controls; after adjusting for potential baseline imbalance, effects were similar but not statistically significant. At endline, differences in ACGG + ATONU children's HAZ and WAZ compared with controls were similar in magnitude to those of ACGG, but not statistically significant. There were no differences in anthropometry between the intervention groups. ACGG + ATONU children had higher dietary diversity and egg consumption than ACGG children at endline. Both interventions showed improvements in chicken management practices. The interventions did not increase anemia, diarrhea, fever, or vomiting, and the ACGG + ATONU group at midline showed reduced risk of fever.

**Conclusions:**

A chicken production intervention with or without nutrition-sensitive BCC may have benefited child nutrition and did not increase morbidity.

This trial was registered at clinicaltrials.gov as NCT03152227.

See corresponding commentary on page 2617.

## Introduction

Globally, stunting affects 159 million children <5 y old, nearly one-quarter of all children. In Ethiopia, ∼38% of children <5 y are stunted and many more experience growth faltering (1). Stunting is a marker of sustained nutritional deficiency and is associated with a number of adverse health outcomes, including compromised cognitive, immune, and metabolic function (2, 3). To address the persistent problem of child undernutrition, nutrition-specific interventions are critical, but are estimated to collectively alleviate the global burden of stunting by only one-fifth (4). Thus, evidence of effective nutrition-sensitive interventions—including improvements in water, sanitation, and hygiene (WASH); agriculture; and women's empowerment—is urgently needed to fill the remaining gap (5, 6).

There has been growing recognition of agriculture's potential to improve undernutrition, because small farms support the livelihoods of 2.5 billion people worldwide and provide 80% of the food supply in Asia and sub-Saharan Africa (7). Still, evidence of the effects of nutrition-sensitive agriculture on child nutrition outcomes remains limited (8). The promotion of animal agriculture has been recognized as a promising strategy for improving nutrition in low-income settings, especially given its potential to address several agriculture–nutrition pathways simultaneously (9–11). These pathways include empowering women (12); providing access to high-quality foods on farms and in markets, especially in rural areas where these foods are scarce and/or expensive (13); and increasing the income available for nutrition and health purchases (9, 14–17). Animal source foods—including meat, eggs, and dairy—have been associated with improved child growth in low-income countries (8, 18–21), likely due to their high micronutrient content and function as a complete source of amino acids. In recent years, chicken production has been highlighted as a scalable nutrition-sensitive intervention, based on its potential to provide a rich and renewable source of micronutrients and income from eggs, and the trend for women to be the primary decision-makers over chicken production (21, 22). However, research has consistently shown that the inclusion of a strong behavior change component is needed in order for agriculture to achieve impacts on nutrition (8). Thus, agricultural interventions might only be expected to improve child nutrition if they incorporate behavior change communication (BCC) that specifically encourages activation of these pathways.

Although animal production could benefit nutrition, simultaneous increases in exposure to fecal contamination by animals may adversely affect child nutrition and health (20, 23, 24). This could be especially true in Ethiopia, where ∼48% of poultry producers keep their chickens indoors overnight (25). Exposure to animal feces may negatively affect child nutritional status through the fecal–oral transmission of harmful zoonotic enteric pathogens, including *Campylobacter, Salmonella* (most common in poultry), *Cryptosporidium,Listeria*, and *Escherichia coli* (most common in ruminants) (26). Chronic exposure to pathogens can lead to a subclinical intestinal condition known as environmental enteric dysfunction, characterized by blunting of gut villi, increased gut permeability, chronic inflammation, impaired nutrient absorption, and growth faltering (27–29).

**FIGURE 1:**
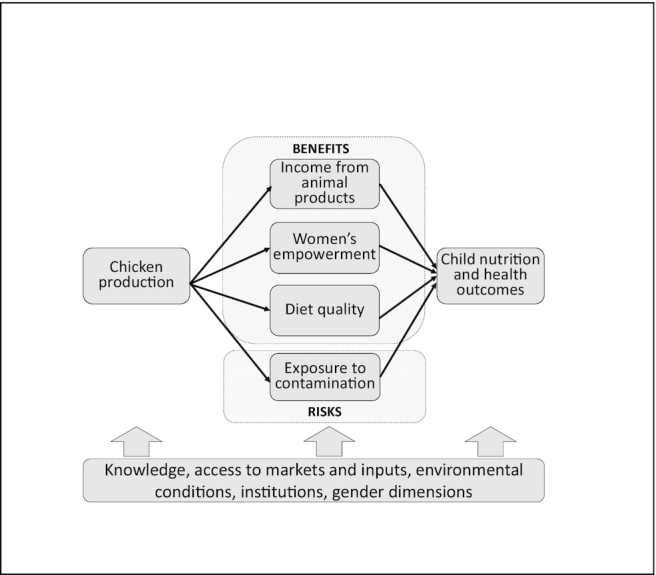
Hypothesized pathways between chicken production and child nutrition and health outcomes.

The conceptual framework in **Figure 1** shows the hypothesized pathways between chicken production and child nutrition and health based on evidence from 2 main areas: *1*) the agriculture–nutrition pathways identified by Herforth and Harris (16), and *2*) the pathways through which exposure to fecal contamination could adversely affect nutrition and health (29–32).

The balance of potential benefits and harms caused by domestic animals has raised questions about whether chicken production should be promoted as a nutrition strategy. Whereas previous research on this topic has largely come from observational data, we assessed the effects of chicken production, with or without a nutrition-sensitive behavior change intervention, on child nutrition and health outcomes through a cluster-randomized trial in rural Ethiopia. We also evaluated the impact of the intervention on intermediaries, including diet, income, women's decision making, and environmental conditions.

## Methods

### Trial design

The African Chicken Genetic Gains (ACGG) project was a cluster-randomized trial (NCT03152227) implemented by the International Livestock Research Institute to evaluate high-yielding, tropically adapted chicken genotypes to increase smallholder productivity, improve incomes, empower women farmers, and identify the genotypes preferred by farmers. Villages (clusters) were allocated in equal proportions to receive no intervention, the chicken intervention only (ACGG), or the chicken intervention combined with the nutrition-sensitive [Agriculture to Nutrition (ATONU)] intervention (ACGG + ATONU). The sample size was determined based on detecting an effect on women's dietary diversity scores (Minimum Dietary Diversity for Women, or MDD-W), the primary outcome of the study, which will be assessed in a forthcoming article.

### Participants

Inclusion criteria for participating households were having produced chickens for ≥2 y, having <50 birds, having ≥1 woman of reproductive age (18–49 y at enrollment), and providing informed consent. One index child was enrolled in the study if there was a child aged 0–35 mo living in the household at baseline, midline, or endline. The trial was implemented in rural agricultural villages in 4 regions of Ethiopia: Amhara; Oromia; Southern Nations, Nationalities, and Peoples’ Region; and Tigray.

### Interventions

As part of the ACGG intervention, 5 chicken breeds were tested and delivered to farmers, including Sasso-RIR, Kuroiler, Sasso, Horro, and Koekoek. These improved varieties produce ≥200% more eggs than local breeds (33). Each household received 25 vaccinated chicks that were ∼6 wk old. To participate, households had to agree to incur the costs of providing chickens with night shelter, daytime enclosures or partitions, feed supplementation, and additional vaccinations. Households were advised to use a semi-scavenging system, in which chickens are let out for several hours twice per day to forage for freely available food in the environment, and to provide birds with supplemental feed. Actual chicken husbandry practices were implemented by farmers according to their individual preferences.

The ACGG + ATONU arm included an additional nutrition-sensitive component targeting the woman of reproductive age enrolled. This component included the promotion of homegardening, the provision of fruit and vegetable seeds for the gardens, and BCC. The BCC topics included the importance of WASH for health and nutrition, emphasizing handwashing, sanitation, and hygiene concerns related to chicken production; women's empowerment in household decision making and budgeting, and the importance of male engagement; and child feeding practices that promoted chicken products as part of a diverse diet containing fruits and vegetables. The nutrition-sensitive interventions were delivered by a behavior change specialist with visual aids through group and individual meetings with women over a 14-mo period both with and without male household heads, from February 2017 to April 2018. On average, participants in the ACGG + ATONU arm reported attending 4.3 sessions, although with a high SD of 6.8 sessions. Seeds for the homegardening activities were distributed in May 2017 and included carrot, tomato, onion, lettuce, cabbage, swiss chard, beetroot, hot pepper, and watermelon.

### Randomization sequence and allocation

Four rural regions were purposively selected by the ACGG program based on their suitable agroecologies for chicken production. Twenty districts were selected from these regions using the same agroecological criteria. All districts deemed suitable were listed and stratified into 3 agroecologies (highland, mid-altitude, and lowland), allowing investigators to evaluate the effectiveness of the intervention and breeds’ viability in different contexts. To select program villages, ACGG created a sampling frame of villages within these districts that met their criteria of geographic diversity, poultry producing capacity, and number of smallholder households producing chickens. Forty villages were randomly selected to participate in the ACGG program and all villages agreed to participate. The 20 ACGG + ATONU villages were randomly selected from the ACGG villages. The 20 non-ACGG villages forming the control group were randomly drawn from the same sampling frame used by ACGG.

During the baseline survey, 40 households were screened in each of the 40 ACGG villages, and ∼35 households/village meeting the inclusion criteria were enrolled. From the 20 selected control villages, ∼50 households/village were screened and ∼35 were enrolled. If >40 households/village met the criteria, a simple random lottery system was applied in both the intervention and control villages. There were ≥35 households that met the inclusion criteria in all cases. After screening and selection, 6 households did not consent to participate. In total, 2658 households were screened and 2117 households were ultimately enrolled.

The nature of the intervention made it impossible to blind the study participants and investigators to their treatment status. However, participants were not explicitly told which study arm they were in and control groups were not informed of the intervention arms.

### Data collection and outcome assessment

Data were collected using Open Data Kit on tablets during 3 household visits at baseline (November 2016), midline (July 2017), and endline (April 2018). The questionnaire concerning chicken production and household nutrition and health was administered to the woman of reproductive age enrolled in the study, whereas a household questionnaire on assets, agricultural production, and household demographics was administered to the (male or female) household head. All participants were followed up at midline (9 mo) and endline (18 mo). Field teams conducted interviews and assessed child height and weight. Anthropometry measures were taken twice for each individual by trained enumerators. The weight of the children was measured to the nearest 0.1 kg using the UNICEF electronic scale. Recumbent length and height were measured to the nearest 0.1 cm using UNICEF's recommended model wooden board, as per the WHO/UNICEF protocol (34).

The outcomes hypothesized to be intermediaries on the child nutrition pathway included number of days the child consumed eggs in the previous week, number of local chickens owned, number of improved chickens owned, relative income from chickens in the previous week (as a proportion of median annual household expenditures in the village), child's dietary diversity score in the previous 7 d [using the categories for the minimum dietary diversity for children (35)], a score of women's input into decision making related to chickens [adapted from the Women's Empowerment in Agriculture Index (36)], and the number of eggs produced in the household the previous week. We measured frequency of egg consumption and child dietary diversity over 7 d instead of over 1 d because our baseline data showed that eggs were not consumed daily in most households. The score of women's decision making regarding chickens was derived using household survey questions about the extent of a woman's input into chicken production, chicken use, use of eggs for home consumption, marketing of eggs, slaughter of chickens for home consumption, and marketing of chickens. The score was then constructed based on the proportion of these 6 activities for which women responded that they had input into some, most, or all decisions.

Contamination from animals was measured using the following 6 variables, as well as a summary score that is a count of these practices (from 0 to 6): household had a chicken coop; household has a chicken coop separate from the household; household has a chicken coop where animals are enclosed; chickens do not roam freely at night; chickens did not sleep in the house last night; and no visible feces are present in/around the household. The binary variables related to features of the chicken coop and presence of feces were based on enumerator observations, whereas whether chickens slept in the house the previous night or roamed freely at night were based on survey questions asked of the woman respondent.

### Statistical methods

The interventions were coded using 2 binary variables indicating whether they were in the ACGG or ACGG + ATONU clusters. The child nutrition and health outcomes analyzed included height-for-age *z* scores (HAZ), weight-for-age *z* scores (WAZ), and weight-for-height *z* scores (WHZ) [as measured by WHO 2006 growth charts (37)]; child anemia (defined as altitude-adjusted hemoglobin <11 g/dL); and fever, vomiting, and diarrhea in the past 2 wk reported by caregivers. Child anthropometric *z* scores were treated as missing if they were outside of the feasible range of <−6 or >6. Across all 3 time points and study arms, this amounted to 98 records for HAZ, 47 for WAZ, and 23 for WAZ. We used ordinary least squares (OLS) to assess the effect of the interventions on the outcomes of HAZ, WAZ, and WHZ at midline and endline. We also used OLS for the exploratory outcomes of the number of local chickens owned, the number of improved chickens owned, the score of women's decision making in chicken production, 7-d frequency of egg consumption (number of days), 7-d child dietary diversity, number of eggs produced in the previous week, and the overall chicken management score. For the binary outcomes of diarrhea, vomiting, fever, and anemia, and the 6 binary measures of chicken management practices, we used log binomial models to estimate risk ratios. Poisson distributions were used in place of binomial distributions in cases of nonconvergence.

In supplemental analyses, we tested for modification of the interventions’ effects on child nutrition and health outcomes by child age and sex, and by baseline chicken management score (binary high or low, defined as above or below the median score), using a Wald chi-square test (see **Supplemental Tables 1** and **2**) in unadjusted regressions.

All models were adjusted for clustering at the village level and for the baseline value of the outcome variable. Because there were only 20 clusters in each study arm, all CIs were bootstrapped with 1000 iterations. Adjusted models controlled for baseline household characteristics of region, wealth quintile (using the first component of a principal component analysis of assets owned), number of other livestock owned, number of household members, the woman's years of education, the woman's age, having improved water [yes/no, based on the WHO/UNICEF definition (38)], having improved sanitation [yes/no, based on the WHO/UNICEF definition (38)], child age group (0–11, 12–23, and 24–35 mo), and sex of the index child (equal to 1 if the child was female). All analyses were conducted using Stata 16 (StataCorp LLC).

### Ethics statement

This research obtained ethical approval from the Harvard Office of Human Research Administration (United States) and the institutional review board of the Addis Continental Institute of Public Health (Ethiopia) in adherence to the principles of the Declaration of Helsinki as revised in 1983.

## Results

**FIGURE 2:**
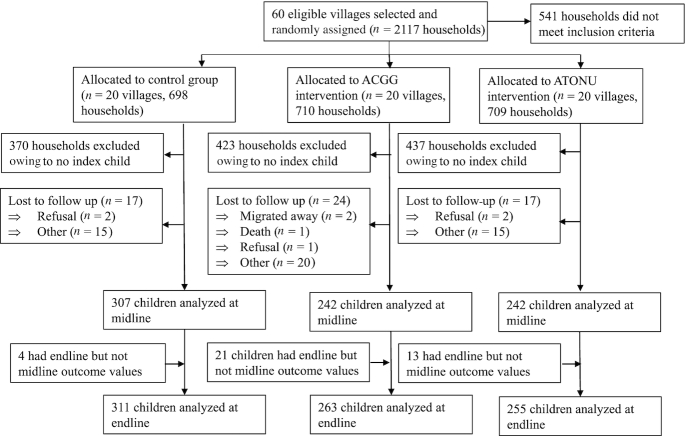
Participant flow diagram. ACGG, African Chicken Genetic Gains; ATONU, Agriculture to Nutrition.

**Figure 2** describes the flow of study participants. The final analytic sample contained 829 children who had baseline and endline data. Eleven households with index children were lost to follow-up at midline (attrition rate = 1.3%) and all but 2 were recovered at endline. On average, households with an index child had younger woman respondents (by ∼5 y), were slightly larger (by 0.6 members), and had 0.5 more local chickens at baseline than households without an index child (data not shown).

**Table 1** shows baseline characteristics, stratified by the 3 study arms. There is some indication that the ACGG and ACGG + ATONU groups had slightly greater wealth, a greater number of other livestock owned, more years of education among women of reproductive age, and a higher proportion of households with improved water and sanitation.

**TABLE 1:** Baseline characteristics of randomly assigned children and their households by randomly assigned group^1^

	Control (*n* = 311)	ACGG intervention (*n* = 265)	ACGG + ATONU intervention (*n* = 255)
Child age at enrollment, mo			
0–12	103 (33.1)	81 (30.8)	77 (30.2)
>12–24	119 (38.3)	88 (33.5)	101 (39.6)
>24–36	89 (28.6)	94 (35.7)	77 (30.2)
Female child	163 (52.4)	140 (53.2)	131 (51.4)
Lowest wealth quintile	78 (25.1)	41 (15.6)	42 (16.5)
Highest wealth quintile	47 (15.1)	64 (24.3)	62 (24.3)
Woman age, y	29.7 ± 5.6	31.7 ± 6.3	31.5 ± 6.4
Household members, *n*	6.2 ± 2.0	6.9 ± 1.9	6.8 ± 1.9
Woman schooling, y	3.1 ± 3.3	3.4 ± 3.9	3.5 ± 3.8
Livestock at baseline, *n*	4.7 ± 4.7	7.2 ± 6.0	6.7 ± 5.7
Improved water	252 (81.6)	224 (87.2)	223 (87.8)
Improved sanitation	76 (24.5)	72 (27.4)	90 (35.3)

1 Values are *n* (%) for binary/categorical variables and means ± SDs for continuous variables. ACGG, African Chicken Genetic Gains; ATONU, Agriculture to Nutrition.

**Table 2** shows the effect of the interventions on HAZ, WHZ, and WAZ at midline and endline. At midline, the ACGG + ATONU group had significantly higher HAZ than the control group in unadjusted analyses. No other statistically significant differences in anthropometry (*P* < 0.05) were observed at midline. At endline, children's HAZ in the ACGG group were 0.28 higher, and WAZ were 0.18 higher, than those of the control group in unadjusted analyses. We did not observe any differences in *z* scores between the ACGG and ACGG + ATONU groups.

**TABLE 2:** Effect of the ATONU intervention on child HAZ, WHZ, and WAZ at midline (9 mo) and endline (18 mo)^1^

				ACGG vs. Control	ACGG/ATONU vs. Control	ACGG/ATONU vs. ACGG	ACGG vs. Control	ACGG/ATONU vs. Control	ACGG/ATONU vs. ACGG
	Control	ACGG	ACGG/ATONU	Unadjusted mean difference (95% CI)	Unadjusted mean difference (95% CI)	Unadjusted mean difference (95% CI)	Adjusted mean difference (95% CI)	Adjusted mean difference (95% CI)	Adjusted mean difference (95% CI)
Midline	*n* = 307	*n* = 242	*n* = 242						
HAZ	−1.63 ± 1.80	−1.56 ± 1.73	−1.34 ± 1.80	0.08 (−0.19, 0.33)	0.28 (0.02, 0.54)**	0.20 (−0.06, 0.46)	0.02 (−0.25, 0.29)	0.22 (−0.00, 0.45)*	0.20 (−0.04, 0.45)
WHZ	0.11 ± 1.31	0.06 ± 1.18	−0.02 ± 1.28	−0.09 (−0.32, 0.14)	−0.19 (−0.44, 0.06)	−0.10 (−0.29, 0.09)	−0.11 (−0.36, 0.14)	−0.21 (−0.44, 0.05)*	−0.09 (−0.29, 0.10)
WAZ	−0.87 ± 1.34	−0.75 ± 1.32	−0.78 ± 1.31	0.10 (−0.10, 0.29)	0.03 (−0.17, 0.23)	−0.07 (−0.25, 0.11)	0.04 (−0.17, 0.25)	−0.04 (−0.26, 0.17)	−0.08 (−0.27, 0.10)
Endline	*n* = 311	*n* = 263	*n* = 255						
HAZ	−1.83 ± 1.47	−1.51 ± 1.73	−1.55 ± 1.70	0.28 (0.05, 0.50)**	0.25 (−0.02, 0.46)*	−0.03 (−0.25, 0.20)	0.21 (−0.02, 0.44)*	0.20 (−0.06, 0.46)	−0.01 (−0.22, 0.19)
WHZ	−0.15 ± 1.18	−0.16 ± 1.19	−0.01 ± 1.40	−0.00 (−0.26, 0.26)	0.15 (−0.15, 0.46)	0.15 (−0.14, 0.45)	−0.03 (−0.28, 0.23)	0.12 (−0.18, 0.42)	0.15 (−0.15, 0.45)
WAZ	−1.17 ± 1.15	−0.98 ± 1.28	−0.92 ± 1.23	0.18 (0.01, 0.36)**	0.23 (−0.03, 0.49)*	0.01 (−0.20, 0.22)	0.14 (−0.04, 0.32)	0.15 (−0.08, 0.38)	0.01 (−0.22, 0.23)

1 Values are means ± SDs or mean differences (95% CIs) unless otherwise indicated. Robust bootstrapped CIs are clustered at the village level. ^*,**^Significant difference: *P < 0.10, **P < 0.05. Unadjusted and adjusted analyses controlled for the baseline child *z* score. Adjusted regressions included the following baseline variables: age category of child, sex of child, wealth quintiles, number of other livestock, number of household members, years of education of mother, maternal age, having improved water, and having improved sanitation. ACGG, African Chicken Genetic Gains; ATONU, Agriculture to Nutrition; HAZ, height-for-age *z* score; WAZ, weight-for-age *z* score; WHZ, weight-for-height *z* score.

In supplemental analyses, we found evidence of effect modification of the ACGG + ATONU intervention by age group at midline for WHZ and WAZ, and at endline for WAZ, such that effects increased with age. We observed no effect modification by high or low baseline chicken management score or sex on HAZ, WHZ, and WAZ (Supplemental Table 1).

**Tables 3** and **4** present the effect of the interventions on hypothesized intermediaries of nutrition, including chicken management practices. At midline, there were improvements in intermediaries within both interventions. The ACGG and ACGG + ATONU intervention groups had higher levels of improved chickens, women's decision making over chicken production, frequency of children's egg consumption in the previous week, and egg production in the previous week than the control group at midline (*P* values < 0.05). At endline effects were similar to midline, and both intervention groups had a higher number of improved chickens, increases in income from chicken production, and increases in egg production compared with control households. At endline, the ACGG + ATONU group had higher levels of women's empowerment in chicken production and frequency of children's egg consumption than the control group, and children in the ACGG + ATONU group ate eggs ∼0.4 more times and ate 0.5 more food groups in the last week than children in the ACGG group.

**TABLE 3:** Effect of ACGG and ACGG/ATONU interventions on chicken management practices at midline (9 mo)^1^

				ACGG vs. Control	ACGG/ATONU vs. Control	ACGG/ATONU vs. ACGG	ACGG vs. Control	ACGG/ATONU vs. Control	ACGG/ATONU vs. ACGG
	Control *n* = 307	ACGG *n* = 242	ACGG/ATONU *n* = 242	Unadjusted RR or mean difference (95% CI)	Unadjusted RR or mean difference (95% CI)	Unadjusted RR or mean difference (95% CI)	Adjusted RR or mean difference (95% CI)	Adjusted RR or mean difference (95% CI)	Adjusted RR or mean difference (95% CI)
Program implementation indicators									
Local chickens, *n*	2.5 ± 3.9	2.3 ± 3.7	2.2 ± 3.7	−0.18 (−0.98, 0.62)	−0.53 (−1.29, 0.23)	−0.36 (−1.01, 0.30)	−0.44 (−1.21, 0.33)	−0.73 (−1.43, 0.04)**	−0.29 (−0.96, 0.37)
Improved chickens, *n*	1.2 ± 4.6	7.6 ± 8.4	7.7 ± 8.5	6.25 (4.35, 8.15)***	6.36 (4.20, 8.51)***	0.11 (−2.42, 2.64)	6.92 (4.89, 8.96)***	6.82 (4.74, 8.90)***	−0.10 (−2.46, 2.25)
Women's decision making related to chickens, out of 6	0.3 ± 0.4	0.4 ± 0.4	0.4 ± 0.4	0.11 (0.02, 0.20)**	0.12 (0.04, 0.21)***	0.01 (−0.09, 0.11)	0.09 (0.01, 0.16)**	0.11 (0.03, 0.18)***	0.02 (−0.06, 0.10)
Chicken income, proportion of expenditure	0.1 ± 0.3	0.1 ± 0.1	0.1 ± 0.2	−0.02 (−0.07, 0.04)	−0.02 (−0.07, 0.03)	−0.01 (−0.04, 0.03)	−0.02 (−0.07, 0.03)	−0.02 (−0.07, 0.03)	−0.00 (−0.03, 0.03)
Frequency of child's egg consumption last week, *n* days	0.3 ± 0.9	0.6 ± 1.2	0.8 ± 1.7	0.34 (0.07, 0.60)**	0.55 (0.23, 0.88)***	0.21 (−0.15, 0.58)	0.22 (−0.04, 0.47)*	0.45 (0.16, 0.75)***	0.24 (−0.10, 0.58)
Child's 7-d dietary diversity, categories	3.6 ± 1.6	4.0 ± 1.6	3.8 ± 1.9	0.38 (0.09, 0.68)**	0.20 (−0.41, 0.80)	−0.18 (−0.76, 0.39)	0.16 (−0.17, 0.49)	0.00 (−0.47, 0.47)	−0.16 (−0.63, 0.31)
Eggs produced in household last week, *n*	3.6 ± 7.5	7.1 ± 11.6	7.1 ± 15.0	3.55 (0.97, 6.12)***	3.43 (0.92, 5.95)***	−0.11 (−3.00, 2.74)	3.27 (0.82, 5.73)***	3.05 (0.74, 5.35)***	−0.23 (−3.03, 2.58)
Chicken management practices									
Has coop, %	48.5 (149)	74.8 (181)	70.7 (171)	1.52 (1.20, 1.92)***	1.44 (1.13, 1.83)***	0.95 (0.80, 1.13)	1.57 (1.32, 1.87)***	1.45 (1.21, 1.74)***	0.92 (0.82, 1.04)
Has coop that is separated from house, %	26.7 (82)	44.2 (107)	45.5 (110)	1.46 (0.96, 2.18)*	1.45 (0.92, 2.28)	0.99 (0.75, 1.32)	1.67 (1.20, 2.33)***	1.67 (1.18, 2.36)***	1.00 (0.77, 1.30)
Has enclosed coop, %	15.6 (48)	33.9 (82)	30.6 (74)	2.12 (1.34, 3.35)***	1.94 (1.20, 3.15)***	0.92 (0.64, 1.32)	2.05 (1.33, 3.15)***	1.83 (1.15, 2.91)**	0.90 (0.66, 1.22)
Chickens do not roam freely at night, %	35.8 (110)	42.6 (103)	38.8 (94)	1.21 (0.94, 1.57)	1.11 (0.85, 1.46)	0.92 (0.69, 1.24)	1.24 (0.97, 1.58)*	1.12 (0.87, 1.42)	0.90 (0.69, 1.17)
Chickens did not sleep in house last night, %	63.2 (194)	70.2 (170)	70.7 (171)	1.01 (0.88, 1.14)	1.02 (0.87, 1.18)	1.01 (0.87, 1.19)	1.03 (0.89, 1.20)	1.06 (0.90, 1.24)	1.02 (0.87, 1.21)
No visible animal feces on compound, %	41.0 (126)	33.9 (82)	33.5 (81)	0.81 (0.61, 1.08)	0.80 (0.59, 1.09)	0.99 (0.69, 1.41)	0.87 (0.66, 1.13)	0.86 (0.64, 1.17)	1.00 (0.72, 1.38)
Chicken management score	2.3 ± 1.4	3.0 ± 1.5	2.9 ± 1.7	0.59 (0.14, 1.04)***	0.49 (−0.00, 0.99)*	−0.10 (−0.59, 0.40)	0.65 (0.30, 1.01)***	0.54 (0.13, 0.94)***	−0.11 (−0.54, 0.31)

1 Values are % (*n*) for binary variables and mean ± SDs for continuous variables in columns 2–4. Values are RRs (95% CIs) for binary variables or mean differences (95% CIs) for continuous variables in columns 5–10. Robust bootstrapped CIs are clustered at the village level. ^*,**,***^Significant difference: *P < 0.1, **P < 0.05, ***P < 0.01. Adjusted regressions include the following baseline variables: wealth quintiles, number of other livestock, number of household members, years of education of mother, maternal age, having improved water, and having improved sanitation. The 6 variables comprising the chicken management score are as follows: has a chicken coop, has a coop that is separated from the house, has an enclosed coop, chickens do not roam freely at night, chickens did not sleep in the house last night, and no visible animal feces on the compound. ACGG, African Chicken Genetic Gains; ATONU, Agriculture to Nutrition; RR, risk ratio.

**TABLE 4:** Effect of ACGG and ACGG/ATONU interventions on chicken management practices at endline (18 mo)^1^

				ACGG vs. Control	ATONU vs. Control	ACGG/ATONU vs. ACGG	ACGG vs. Control	ATONU vs. Control	ACGG/ATONU vs. ACGG
	Control (*n* = 311)	ACGG (*n* = 263)	ACGG/ATONU (*n* = 255)	Unadjusted RR or mean difference (95% CI)	Unadjusted RR or mean difference (95% CI)	Unadjusted RR or mean difference (95% CI)	Adjusted RR or mean difference (95% CI)	Adjusted RR or mean difference (95% CI)	Adjusted RR or mean difference (95% CI)
Program implementation indicators									
Local chickens, *n*	2.1 ± 4.3	1.8 ± 3.2	1.6 ± 2.5	−0.36 (−1.23, 0.52)	−0.73 (−1.60, 0.14)*	−0.37 (−0.98, 0.23)	−0.68 (−1.46, 0.11)*	−0.85 (−1.66, −0.04)**	−0.18 (−0.70, 0.35)
Improved chickens, *n*	1.2 ± 2.9	4.0 ± 5.3	3.1 ± 4.0	2.76 (1.33, 4.20)***	1.86 (0.83, 2.89)***	−0.90 (−2.33, 0.52)	3.05 (1.79, 4.31)***	2.03 (1.13, 2.93)***	−1.02 (−2.22, 0.18)*
Women's decision making related to chickens, out of 6	0.3 ± 0.4	0.4 ± 0.4	0.4 ± 0.4	0.06 (−0.02, 0.14)	0.10 (0.00, 0.20)**	0.04 (−0.06, 0.13)	0.05 (−0.03, 0.13)	0.10 (−0.00, 0.20)*	0.04 (−0.06, 0.15)
Chicken income, proportion of expenditure	0.1 ± 0.2	0.2 ± 0.3	0.1 ± 0.3	0.10 (0.03, 0.16)***	0.07 (0.01, 0.12)**	−0.03 (−0.10, 0.04)	0.10 (0.04, 0.16)***	0.07 (0.02, 0.12)***	−0.03 (−0.09, 0.03)
Frequency of child's egg consumption last week, *n* days	0.6 ± 1.2	0.8 ± 1.6	1.3 ± 1.9	0.21 (−0.07, 0.49)	0.68 (0.26, 1.10)***	0.47 (0.05, 0.90)**	0.18 (−0.09, 0.46)	0.62 (0.27, 0.98)***	0.44 (0.04, 0.84)**
Child's 7-d dietary diversity, categories	4.0 ± 1.8	3.8 ± 2.1	4.3 ± 1.9	−0.19 (−0.69, 0.30)	0.33 (−0.12, 0.77)	0.52 (0.02, 1.01)**	−0.32 (−0.87, 0.22)	0.18 (−0.23, 0.60)	0.51 (0.05, 0.96)**
Eggs produced in household last week, *n*	5.6 ± 11.8	10.7 ± 12.5	9.1 ± 13.4	5.17 (2.00, 8.34)***	3.32 (0.28, 6.35)**	−1.85 (−5.38, 1.68)	4.63 (1.80, 7.46)***	2.70 (0.09, 5.30)**	−1.94 (−4.94, 1.07)
Chicken management practices									
Has coop, %	40.5 (126)	65.4 (172)	62.7 (160)	1.57 (1.22, 2.07)***	1.54 (1.19, 1.98)***	0.98 (0.81, 1.18)	1.54 (1.29, 1.84)***	1.48 (1.22, 1.79)***	0.96 (0.85, 1.08)
Has coop that is separated from house, %	22.5 (70)	42.2 (111)	40.8 (104)	1.77 (1.15, 2.73)***	1.70 (1.10, 2.64)**	0.96 (0.72, 1.27)	1.89 (1.40, 2.55)***	1.79 (1.31, 2.43)***	0.95 (0.76, 1.18)
Has enclosed coop, %	15.1 (47)	31.2 (82)	31.4 (80)	2.00 (1.30, 3.08)***	2.05 (1.32, 3.19)***	1.03 (0.74, 1.42)	1.92 (1.39, 2.67)***	1.99 (1.41, 2.82)***	1.03 (0.80, 1.33)
Chickens do not roam freely at night, %	38.6 (120)	40.3 (106)	42.0 (107)	1.04 (0.78, 1.39)	1.09 (0.83, 1.43)	1.04 (0.81, 1.33)	1.01 (0.77, 1.31)	1.06 (0.85, 1.31)	1.05 (0.86, 1.29)
Chickens did not sleep in house last night, %	66.2 (206)	73.0 (192)	74.9 (191)	1.04 (0.89, 1.21)	1.08 (0.95, 1.23)	1.04 (0.92, 1.18)	1.04 (0.91, 1.20)	1.09 (0.96, 1.25)	1.05 (0.94, 1.17)
No visible animal feces on compound, %	47.3 (147)	39.5 (104)	40.8 (104)	0.85 (0.66, 1.09)	0.86 (0.66, 1.13)	1.02 (0.73, 1.41)	0.90 (0.71, 1.13)	0.91 (0.72, 1.17)	1.02 (0.79, 1.32)
Chicken management score	2.3 ± 1.4	2.9 ± 1.6	2.9 ± 1.7	0.54 (0.12, 0.95)**	0.55 (0.16, 0.93)***	0.01 (−0.35, 0.36)	0.55 (0.25, 0.84)***	0.56 (0.28, 0.85)***	0.01 (−0.24, 0.27)

1 Values are % (*n*) for binary variables and means ± SDs for continuous variables in columns 2–4. Values are RRs (95% CIs) for binary variables and mean differences (95% CIs) for continuous variables in columns 5–10. Robust bootstrapped CIs are clustered at the village level. ^*,**,***^Significant difference: *P < 0.1, **P < 0.05, ***P < 0.01. Adjusted regressions include the following baseline variables: wealth quintiles, number of other livestock, number of household members, years of education of mother, maternal age, having improved water, and having improved sanitation. The 6 variables comprising the chicken management score are as follows: has a chicken coop, has a coop that is separated from the house, has an enclosed coop, chickens do not roam freely at night, chickens did not sleep in the house last night, and no visible animal feces on the compound. ACGG, African Chicken Genetic Gains; ATONU, Agriculture to Nutrition; RR, risk ratio.

Tables 3 and 4 demonstrate an increased use of chicken management practices hypothesized to limit exposure to contamination in the intervention groups. At midline and endline, both intervention groups were more likely to have a chicken coop, have a coop where chickens were confined, and have a coop separated from the household. At midline, ACGG group households were less likely to let their chickens roam freely at night than control households. Overall, there was an increase in the chicken management score by ∼0.6 practices in both intervention arms relative to the control group at endline. There were no significant differences in management practices between the ACGG and ACGG + ATONU arms.

As **Table 5** shows, the ACGG + ATONU group exhibited a reduced risk of fever in the previous 2 wk compared with both the control group and the ACGG group at midline. Otherwise, there were no significant differences in morbidity outcomes—including fever, vomiting, and diarrhea in the past 2 wk—by treatment group, and no differences in anemia. We saw no differences in hospital or clinic visits across arms (data not shown). Supplemental analyses showed a lower risk of vomiting in children aged 0–12 or 12–24 mo than in those aged 24–36 mo in the ACGG + ATONU intervention group. We observed no effect modification of the interventions by chicken management score or sex for other morbidity outcomes (Supplemental Table 2).

**TABLE 5:** Effect of the ACGG intervention on maternal-reported morbidity and child anemia at midline (9 mo) and endline (18 mo)^1^

				ACGG vs. Control	ACGG/ATONU vs. Control	ACGG/ATONU vs. ACGG	ACGG vs. Control	ACGG/ATONU vs. Control	ACGG/ATONU vs. ACGG
	Control	ACGG	ACGG/ATONU	Unadjusted RR (95% CI)	Unadjusted RR (95% CI)	Unadjusted RR (95% CI)	Adjusted RR (95% CI)	Adjusted RR (95% CI)	Adjusted RR (95% CI)
Midline	*n* = 307	*n* = 242	*n* = 242						
Fever	19.8 (60)	21.5 (52)	11.9 (27)	1.05 (0.69, 1.60)	0.59 (0.38, 0.92)**	0.56 (0.35, 0.92)**	1.06 (0.76, 1.46)	0.62 (0.38, 1.00)**	0.59 (0.35, 0.96)**
Vomiting	9.9 (30)	13.2 (32)	10.6 (24)	1.32 (0.73, 2.39)	1.06 (0.61, 1.86)	0.80 (0.50, 1.28)	1.41 (0.78, 2.53)	1.18 (0.70, 1.98)	0.84 (0.51, 1.37)
Diarrhea	18.2 (55)	15.3 (37)	16.4 (37)	0.85 (0.57, 1.27)	0.90 (0.59, 1.36)	1.05 (0.70, 1.58)	0.80 (0.51, 1.25)	0.84 (0.49, 1.42)	1.05 (0.66, 1.68)
Endline	*n* = 311	*n* = 263	*n* = 255						
Anemia (Hb <11 g/dL)	54.5 (84)	58.3 (70)	57.7 (64)	1.11 (0.85, 1.45)	1.06 (0.84, 1.32)	0.95 (0.72, 1.26)	1.18 (0.89, 1.57)	1.08 (0.86, 1.37)	0.92 (0.69, 1.22)
Fever	15.1 (47)	14.1 (37)	14.5 (37)	0.92 (0.59, 1.41)	0.96 (0.64, 1.44)	1.05 (0.71, 1.55)	0.87 (0.53, 1.42)	0.90 (0.59, 1.36)	1.03 (0.68, 1.58)
Vomiting	7.4 (23)	6.8 (18)	3.9 (10)	0.91 (0.50, 1.68)	0.53 (0.24, 1.16)	0.58 (0.27, 1.25)	1.06 (0.55, 2.02)	0.61 (0.17, 2.16)	0.57 (0.16, 2.07)
Diarrhea	10.0 (31)	12.2 (32)	10.6 (27)	1.22 (0.68, 2.21)	1.05 (0.54, 2.05)	0.86 (0.49, 1.52)	1.27 (0.70, 2.32)	1.11 (0.57, 2.14)	0.87 (0.47, 1.61)

1 Values are the group prevalence based on the % (*n*) in columns 2–4. Values are RRs (95% CIs) in columns 5–10. Outcomes are maternal-reported illnesses for index children in the previous 2 wk. Regressions are log binomial regressions, or Poisson in the case of nonconvergence. Robust bootstrapped CIs are clustered at the village level. Adjusted regressions include the following baseline variables: age category of child, sex of child, wealth quintiles, number of other livestock, number of household members, years of education of mother, maternal age, having improved water, and having improved sanitation. Child anemia data were not collected at midline. Hemoglobin values were adjusted for altitude. **Significant difference: *P* < 0.05. ACGG, African Chicken Genetic Gains; ATONU, Agriculture to Nutrition; RR, risk ratio.

## Discussion

We found that a chicken production intervention in rural Ethiopia with or without a nutrition BCC and homegardening component showed benefits for participants, as measured by several nutrition, health, and intermediary indicators. Relative to the control group, children in the ACGG + ATONU group had higher HAZ at midline, and children in the ACGG group had higher HAZ and WAZ at endline. We did not observe statistically significant differences in anthropometry between the ACGG + ATONU group and either the ACGG or control group at endline. Both interventions showed similar improvements in chicken management practices at midline and endline, and in hypothesized pathways through which chicken production could improve nutrition, including the number of improved chickens owned, egg production, income from chickens, women's empowerment in chicken production, and the index child's frequency of egg consumption. We did not observe harmful effects of the intervention on child morbidity and health, including fever, vomiting, and diarrhea in the past 2 wk, or anemia; although the ACGG + ATONU group showed protective effects against fever at midline.

One explanation for the higher HAZ in both intervention groups and higher WAZ in the ACGG group is that increased egg production could benefit both diets and income—especially the income of women, who tend to control cash from egg sales in Ethiopia (39). Eggs are a rich source of both macro- and micronutrients that are important for early childhood growth and development. They contain a complete source of amino acids, essential fatty acids such as DHA (22:6n–3) that are important for early brain and visual development, and choline, which is an important precursor for the development of phospholipids needed for cellular growth, division, and membrane signaling (40–42). A recent randomized controlled trial in Ecuador found that the consumption of 1 egg/d by children aged 6–9 mo was associated with a reduction in the prevalence of stunting by 47% [prevalence ratio (PR): 0.53; 95% CI: 0.37, 0.77)] and underweight by 74% (PR: 0.26; 95% CI: 0.10, 0.70) (21), potentially due to improvements in DHA and choline (42). Replication of this study in Malawi did not demonstrate improvements for linear growth—which might be due to the fact that fish consumption was already high among the study population—although there were benefits for head circumference (43). These results support the biological plausibility of our findings, especially among a population with a low baseline consumption of animal source foods. The magnitude of the effect on HAZ associated with the intervention arms—of ∼0.2–0.3 SDs—is comparable with effect sizes ranging from ∼0.22 to 0.39 observed with complementary feeding promotion (4).

As for the income pathway, both intervention groups earned ∼$11 more from chickens per week at endline than the control group—a notable amount when mean monthly food expenditures were ∼$23. In the ACGG group, egg production and income from chickens increased at endline relative to midline, although children's diets did not improve. This could mean that households chose to sell their eggs instead of feeding them to children, possibly because eggs can command a high market price due to the high social value of chicken products in Ethiopia (39, 44). Research from Zambia has similarly shown that improving agricultural productivity and access to diverse foods might not always be sufficient to affect children's diets (45, 46); unlike that study, however, we observe an impact on chronic but not acute malnutrition, which suggests that the income pathway had a particularly strong effect in our study. There is substantial literature showing that increasing women's control over resources is associated with higher expenditures on food, health care, and education for children—a result that may have been a factor in this case (47).

Our results showed an additional benefit of the nutrition BCC for children's egg consumption and dietary diversity in the ACGG + ATONU group relative to the ACGG intervention alone. This is consistent with research finding benefits of nutrition behavior change for diets, although not always for child linear growth (8, 46). It is also supported by a number of studies showing the benefits of homestead food production for improving dietary diversity and egg consumption (48), although the magnitude of the dietary effects was smaller in our study by comparison. Evidence of a stronger dietary pathway in the ACGG + ATONU group is further supported by our qualitative endline data (not yet published), which showed a preference for selling eggs in the ACGG group, but feeding eggs to children and family members in the ACGG + ATONU group. The fact that the ACGG + ATONU intervention showed improvements in diets, but only midline and not endline anthropometry, could be due to a lack of effect, seasonality, a lack of power, and/or insufficient follow-up. Because the behavior change group sessions were implemented from February 2017 to April 2018, and endline data were collected beginning in April 2018, the follow-up time could have been inadequate to fully observe the effects of the BCC on child anthropometry, whereas the income effects could have been realized more immediately. Recent evidence from Ethiopia showed that an intensive behavior change intervention incorporating women's empowerment and agricultural activities improved child minimum dietary diversity, and increased HAZ by 0.24 after 2 y (49). As their study and others have described, it often takes 2 y to observe impacts of nutrition-sensitive interventions on child anthropometry and, thus, impacts on dietary indicators should be prioritized (8).

In contrast with previous evidence showing harmful effects of homestead chicken production on WASH conditions, we found no evidence that the intervention worsened the WASH environment, nor that it adversely affected child health or nutrition outcomes. In fact, we found that children in the ACGG + ATONU group had a lower risk of fever at midline, suggesting either improved immunity or protection from the BCC intervention. The household survey data showed no differences between study arms in hospital or clinic visits, which further supports our morbidity results. There are several possible explanations for these findings. First, the use of a chicken coop, which was promoted among the intervention group but not universally adopted, could have been effective in limiting the exposure of young children to contamination. Another explanation is that the amount of contamination in these household environments is ubiquitous and high, making a small increase or decrease insufficient to result in measurable health impacts. The latter explanation is consistent with findings from 3 recent trials, which found no impacts of WASH interventions on child nutritional status and hypothesized that the lack of effects may have been due to the persistently high degree of environmental exposures even after successful behavior change occurred (50, 51). Although we did not measure pathogens directly, our data showed that more than half of households had animal feces visible on the property in all groups at all time points, and that the intervention had no measurable effect on this variable despite changes in the chicken management practices we measured. Although the ACGG intervention promoted certain husbandry practices like use of a separated chicken coop, it did not enforce them or promote them as a human health intervention. The ACGG + ATONU intervention's curriculum emphasized the importance of environmental contamination and overall WASH for child nutrition and health, but households were not provided with materials to support these behaviors (e.g., soap, clean water, fencing). As Pickering et al. (51) stated in a follow-up to the SHINE (Sanitation Hygiene Infant Nutrition Efficacy) and WASH Benefits trials, future research should focus on identifying interventions that can minimize a child's exposure to contamination more effectively, and measure contamination directly rather than through proxies.

Although we found no negative impacts of chicken production on child nutrition, health, or the WASH environment, this finding should be interpreted with caution. First, this study was not specifically designed to test the effectiveness of the intervention's ability to limit exposure to contamination. Furthermore, chickens are only 1 factor in a household's overall nutrition and WASH environment, and must be considered in the context of other crops and animals, access to safe and adequate WASH services, intrahousehold dynamics, and the broader food environment. A more holistic approach to improving environmental health in agricultural environments may help to optimize child nutrition and health outcomes. Future research, some of which is already underway, should focus on the effectiveness of BCC for improving WASH in the context of animal agriculture (23), the effectiveness of different animal husbandry systems for limiting exposure to infectious agents (52), and the barriers and facilitators to adopting safe animal husbandry practices in resource-limited settings.

This study has several limitations. First, the child health variables rely on the caregiver's recall of the previous 2 wk. This recall could be inaccurate, or the timing of the recall period might not align with the intervention's greatest impact on these outcomes. In addition, these findings may not be generalizable to other populations. The participants came from households in agroecological zones of Ethiopia that were suitable for chicken production, that were already involved in small-scale chicken production, and that may be wealthier on average than non-livestock-owning households in rural areas. Our analyses also included numerous statistical tests, so it is possible that some of our associations were an artifact of multiple testing. Lastly, because the surveys were conducted 9 mo apart, it is possible that our results were driven by seasonal food insecurity. Food security would have been highest at baseline, lowest at midline, and moderate at endline; thus, the intervention might have had the greatest benefit at midline. Owing to the limited follow-up, we might not have been able to adequately capture these effects in our estimates. However, all 3 arms should be equally affected by seasonal variation, and so comparisons between the intervention and control arms should account for these differences.

Several project implementation challenges should be noted in the interpretation of these findings. Owing to supply chain issues, the distribution of chickens took place over ∼1 y, from August 2016 to August 2017, with a median arrival time in March 2017. This means that some households received their chickens before the baseline survey, and some shortly after the midline survey. As a result, we may have underestimated the effects of the intervention (especially at midline) for individuals who received their chickens later. There was also high fatality among the chicken varieties, with ∼4 improved chickens of 25 given remaining at endline. Half of the chicks distributed were males, which were often sold or slaughtered for consumption, and ∼7/household were lost owing to predation or disease. The birds were vaccinated against Newcastle disease, coccidiosis, parasitic worms, and Gumboro disease, but vaccination is not 100% effective, and other common diseases like salmonellosis, fowl cholera, and fowl pox could be at fault (53, 54). These challenges highlight the necessity of providing households involved in animal production with all required inputs, including access to improved animal feeds, housing supplies, markets, and veterinary care—in addition to thorough training and continued support—for poultry rearing to be sustainable and successful.

In conclusion, we found that an animal production intervention and an additional nutrition-sensitive behavior change intervention may have been associated with increased child growth compared with control households. Our results also showed that adding a nutrition-sensitive behavior change component (the ACGG + ATONU intervention) was associated with improved child feeding behaviors as compared with the ACGG intervention alone, and that the BCC improved women's empowerment in chicken production compared with control households. We found no evidence of harmful effects on child morbidity or anemia. Given the multiple pathways through which chicken production could affect nutrition—including women's empowerment, income, diet quality, and WASH—it is possible that small-scale production of chicken and eggs can help supplement household diets and income. However, these systems must be adequately supported with access to inputs, biosafety measures, veterinary services, and markets in order to be sustainable and effective, and should be coupled with BCC activities in order to achieve maximal impacts on children's diets and health.

## Supplementary Material

nxaa181_Supplemental_File

## Notes

Supported by Bill and Melinda Gates Foundation grant OPP1032718 (to WF).

Author disclosures: The authors report no conflicts of interest.

Supplemental Tables 1 and 2 are available from the “Supplementary data” link in the online posting of the article and from the same link in the online table of contents at https://academic.oup.com/jn/.

Abbreviations used: ACGG, African Chicken Genetic Gains; ATONU, Agriculture to Nutrition; BCC, behavior change communication; HAZ, height-for-age *z* scores; OLS, ordinary least squares; PR, prevalence ratio; WASH, water, sanitation, and hygiene; WAZ, weight-for-age *z* scores; WHZ, weight-for-height *z* scores.
