# Supplementary material for: A Chicken Production Intervention and Additional Nutrition Behavior Change Component Increased Child Growth in Ethiopia: A Cluster-Randomized Trial
Source: J Nutr. 2020 Jul 11;150(10):2806–17. doi: 10.1093/jn/nxaa181 (PMC7549301; doi:10.1093/jn/nxaa181)
Supplement: nxaa181_Supplemental_File [file nxaa181_supplemental_file.docx]

Supplemental Table 1: Effect of the ACGG intervention and ACGG/ATONU intervention on height-for-age z-scores, weight-for-height z-scores and weight-for-age z-scores at midline and endline stratified by baseline child age, sex, and chicken management score^1^

|  | Midline | | | | Endline | | | |
| --- | --- | --- | --- | --- | --- | --- | --- | --- |
|  | ACGG | | ACGG / ATONU | | ACGG | | ACGG / ATONU | |
|  | Mean Difference (95% CI) | p-value for effect modification | Mean Difference (95% CI) | p-value for effect modification | Mean Difference (95% CI) | p-value for effect modification | Mean Difference (95% CI) | p-value for effect modification |
| **HAZ by baseline child age** |  | 0.071 |  | 0.567 |  | 0.519 |  | 0.792 |
| 0-12 mo | -0.14 (-0.56 – 0.29) |  | 0.09 (-0.45 – 0.64) |  | 0.04 (-0.55 – 0.63) |  | 0.08 (-0.50 – 0.66) |  |
| > 12-24 mo | -0.05 (-0.24 – 0.15) |  | 0.37 (-0.08 – 0.81) |  | 0.43 (0.11 – 0.75) |  | 0.30 (-0.01 – 0.61) |  |
| > 24-36 mo | 0.38 (-0.08 – 0.83) |  | 0.33 (0.22 – 0.44) |  | 0.29 (-0.07 – 0.65) |  | 0.27 (-0.13 – 0.68) |  |
| **HAZ by baseline chicken management score** |  | 0.678 |  | 0.339 |  | 0.483 |  | 0.259 |
| Low chicken management score | 0.15 (-0.18 – 0.48) |  | 0.13 (-0.21 – 0.48) |  | 0.37 (-0.03 – 0.77) |  | 0.06 (-0.36 – 0.49) |  |
| High chicken management score | 0.04 (-0.33 – 0.41) |  | 0.38 (0.01 – 0.76) |  | 0.18 (-0.09 – 0.46) |  | 0.33 (0.04 – 0.63) |  |
| **HAZ by sex** |  | 0.934 |  | 0.999 |  | 0.980 |  | 0.099 |
| Boys | 0.08 (-0.26 – 0.43) |  | 0.29 (-0.07 – 0.64) |  | 0.27 (-0.04 – 0.57) |  | 0.07 (-0.29 – 0.44) |  |
| Girls | 0.07 (-0.27 – 0.40) |  | 0.29 (0.00 – 0.57) |  | 0.27 (-0.07 – 0.61) |  | 0.44 (0.07 – 0.81) |  |
| **WHZ by baseline child age** |  | 0.244 |  | 0.002 |  | 0.452 |  | 0.169 |
| 0-12 mo | -0.35 (-0.64 - -0.06) |  | -0.55 (-0.99 - -0.10) |  | -0.17 (-0.59 – 0.25) |  | -0.10 (-0.60 – 0.40) |  |
| > 12-24 mo | 0.07 (-0.05 – 0.20) |  | -0.27 (-0.55 – 0.01) |  | 0.02 (-0.42 – 0.46) |  | 0.15 (-0.23 – 0.52) |  |
| > 24-36 mo | -0.02 (-0.38 – 0.34) |  | 0.26 (-0.48 – 1.01) |  | 0.16 (-0.18 – 0.49) |  | 0.42 (-0.02 – 0.87) |  |
| **WHZ by baseline chicken management score** |  | 0.842 |  | 0.738 |  | 0.239 |  | 0.697 |
| Low chicken management score | -0.12 (-0.49 – 0.25) |  | -0.23 (-0.62 – 0.16) |  | -0.12 (-0.43 – 0.19) |  | 0.11 (-0.26 – 0.48) |  |
| High chicken management score | -0.07 (-0.38 – 0.24) |  | -0.16 (-0.42 – 0.10) |  | 0.09 (-0.21 – 0.40) |  | 0.20 (-0.17 – 0.57) |  |
| **WHZ by sex** |  | 0.840 |  | 0.736 |  | 0.357 |  | 0.201 |
| Boys | -0.12 (-0.40 – 0.17) |  | -0.23 (-0.51 – 0.05) |  | 0.09 (-0.21 – 0.39) |  | 0.29 (-0.03 – 0.61) |  |
| Girls | -0.07 (-0.42 – 0.28) |  | -0.15 (-0.56 – 0.26) |  | -0.08 (-0.39 – 0.24) |  | 0.03 (-0.33 – 0.40) |  |
| **WAZ by age at baseline** |  | 0.278 |  | 0.003 |  | 0.552 |  | 0.001 |
| 0-12 mo | -0.12 (-0.72 – 0.49) |  | -0.36 (-0.69 - -0.03) |  | 0.03 (-0.35 – 0.40) |  | -0.18 (-0.56 – 0.20) |  |
| > 12-24 mo | 0.15 (-0.13 – 0.44) |  | 0.10 (-0.24 - 0.44) |  | 0.29 (0.00 – 0.58) |  | 0.27 (-0.01 – 0.55) |  |
| > 24-36 mo | 0.25 (-0.16 – 0.65) |  | 0.32 (0.22 – 0.41) |  | 0.24 (-0.03 – 0.51) |  | 0.46 (0.18 – 0.74) |  |
| **WAZ by chicken management score** |  | 0.840 |  | 0.055 |  | 0.743 |  | 0.297 |
| Low chicken management score | 0.09 (-0.24 – 0.41) |  | -0.18 (-0.47 – 0.11) |  | 0.15 (-0.11 – 0.40) |  | 0.09 (-0.16 – 0.34) |  |
| High chicken management score | 0.13 (-0.12 – 0.37) |  | 0.18 (-0.06 – 0.43) |  | 0.20 (-0.05 – 0.46) |  | 0.26 (-0.05 – 0.57) |  |
| **WAZ by sex** |  | 0.520 |  | 0.646 |  | 0.102 |  | 0.884 |
| Boys | 0.15 (-0.08 – 0.39) |  | -0.02 (-0.29 – 0.25) |  | 0.31 (0.11 – 0.51) |  | 0.21 (-0.05 – 0.48) |  |
| Girls | 0.05 (-0.21 – 0.31) |  | 0.07 (-0.20 – 0.33) |  | 0.07 (-0.17 – 0.31) |  | 0.19 (-0.08 – 0.46) |  |

^1^Values are Mean Difference (95% CI) in columns 2, 4, 6, and 8. The p-value for effect modification is reported in columns 3, 5, 7, and 9. Robust bootstrapped confidence intervals are clustered at the village level. Models adjusted for the baseline value of the z-score. Low or high chicken management score was a binary variable defined as being above or below the median chicken management score at baseline. ACGG=African Chicken Genetic Gains; ATONU=Agriculture to Nutrition; CI=confidence interval; HAZ=height-for-age z-score; WAZ=weight-for-age z-score; WHZ=weight-for-height z-score.

Supplemental Table 2: Effect of the ACGG intervention and ACGG/ATONU intervention on anemia, fever, vomiting, and diarrhea at midline and endline stratified by baseline child age, sex, and chicken management score

|  | Midline | | | | Endline | | | |
| --- | --- | --- | --- | --- | --- | --- | --- | --- |
|  | ACGG | | ACGG / ATONU | | ACGG | | ACGG / ATONU | |
|  | RR (95% CI) | p-value for effect modification | RR (95% CI) | p-value for effect modification | RR (95% CI) | p-value for effect modification | RR (95% CI) | p-value for effect modification |
| **Anemia status by age at baseline** |  |  |  |  |  | 0.683 |  | 0.090 |
| 0-12 mo |  |  |  |  | 1.10 (0.80 – 1.50) |  | 0.93 (0.70 – 1.23) |  |
| > 12-24 mo |  |  |  |  | 1.06 (0.73 – 1.55) |  | 0.90 (0.63 – 1.29) |  |
| > 24-36 mo |  |  |  |  | 1.42 (0.75 – 2.70) |  | 1.72 (0.99 – 2.97) |  |
| **Anemia status by chicken management score** |  |  |  |  |  | 0.692 |  | 0.703 |
| Low chicken management score |  |  |  |  | 1.19 (0.82 – 1.71) |  | 1.11 (0.79 – 1.56) |  |
| High chicken management score |  |  |  |  | 1.09 (0.75 – 1.57) |  | 1.02 (0.73 – 1.43) |  |
| **Anemia status by sex** |  |  |  |  |  | 0.965 |  | 0.978 |
| Boys |  |  |  |  | 1.11 (0.77 – 1.60) |  | 1.05 (0.79 – 1.41) |  |
| Girls |  |  |  |  | 1.12 (0.80 – 1.57) |  | 1.06 (0.78 – 1.44) |  |
| **Fever by age at baseline** |  | 0.416 |  | 1.000 |  | 0.135 |  | 0.186 |
| 0-12 mo | 0.90 (0.49 – 1.63) |  | 0.61 (0.36 – 1.03) |  | 1.27 (0.64 – 2.53) |  | 1.34 (0.68 – 2.64) |  |
| > 12-24 mo | 0.92 (0.44 – 1.94) |  | 0.61 (0.25 – 1.45) |  | 0.64 (0.34 – 1.21) |  | 0.47 (0.18 – 1.21) |  |
| > 24-36 mo | 1.47 (0.74 – 2.92) |  | 0.60 (0.23 – 1.55) |  | 1.05 (0.38 – 2.93) |  | 1.58 (0.67 – 3.76) |  |
| **Fever by chicken management score** |  | 0.224 |  | 0.989 |  | 0.657 |  | 0.730 |
| Low chicken management score | 0.89 (0.52 – 1.52) |  | 0.61 (0.34 – 1.10) |  | 1.05 (0.59 – 1.84) |  | 0.91 (0.53 – 1.56) |  |
| High chicken management score | 1.30 (0.78 – 2.15) |  | 0.61 (0.34 – 1.11) |  | 0.86 (0.46 – 1.62) |  | 1.05 (0.57 – 1.95) |  |
| **Fever by sex** |  | 0.880 |  | 0.221 |  | 0.920 |  | 0.679 |
| Boys | 1.07 (0.64 – 1.81) |  | 0.76 (0.42 – 1.37) |  | 0.90 (0.51 – 1.59) |  | 0.86 (0.46 – 1.62) |  |
| Girls | 1.03 (0.60 – 1.77) |  | 0.45 (0.23 – 0.88) |  | 0.94 (0.49 – 1.79) |  | 1.07 (0.57 – 2.02) |  |
| **Vomiting by age at baseline** |  | 0.960 |  | 0.658 |  | 0.168 |  | 0.009 |
| 0-12 mo | 1.14 (0.48 – 2.72) |  | 1.34 (0.61 – 2.92) |  | 1.45 (0.67 – 3.12) |  | 0.71 (0.15 – 3.33) |  |
| > 12-24 mo | 1.41 (0.37 – 5.39) |  | 1.27 (0.31 – 5.29) |  | 0.35 (0.10 – 1.26) |  | 0.10 (0.03 – 0.32) |  |
| > 24-36 mo | 1.57 (0.09 – 28.17) |  | 0.51 (0.05 – 5.39) |  | 1.39 (0.03 – 72.66) |  | 1.45 (0.01 – 178.11) |  |
| **Vomiting by chicken management score** |  | 0.623 |  | 0.987 |  | 0.130 |  | 0.829 |
| Low chicken management score | 1.11 (0.48 – 2.56) |  | 1.06 (0.50 – 2.23) |  | 1.37 (0.72 – 2.63) |  | 0.48 (0.18 – 1.29) |  |
| High chicken management score | 1.44 (0.42 – 4.98) |  | 1.05 (0.30 – 3.67) |  | 0.55 (0.17 – 1.78) |  | 0.58 (0.11 – 2.95) |  |
| **Vomiting by sex** |  | 0.462 |  | 0.439 |  | 0.278 |  | 0.768 |
| Boys | 1.59 (0.79 – 3.17) |  |  |  | 1.26 (0.56 – 2.85) |  | 0.61 (0.24 – 1.51) |  |
| Girls |  |  |  |  | 0.67 (0.27 – 1.63) |  | 0.47 (0.12 – 1.81) |  |
| **Diarrhea by age at baseline** |  | 0.450 |  | 0.636 |  | 0.963 |  | 0.971 |
| 0-12 mo | 1.09 (0.58 – 2.07) |  | 0.98 (0.48 – 2.01) |  | 1.13 (0.51 – 0.963) |  | 1.16 (0.54 – 2.50) |  |
| > 12-24 mo | 0.50 (0.22 – 1.15) |  | 0.62 (0.33 – 1.19) |  | 1.10 (0.37 – 3.29) |  | 0.97 (0.32 – 2.87) |  |
| > 24-36 mo | 1.39 (0.05 – 36.43) |  | 1.62 (0.03 – 88.08) |  | 1.87 (0.04 – 86.37) |  | 1.12 (0.04 – 31.15) |  |
| **Diarrhea by chicken management score** |  |  |  |  |  | 0.729 |  | 0.804 |
| Low chicken management score | 0.85 (0.43 – 1.68) | 0.945 | 0.82 (0.41 – 1.64) | 0.666 | 1.43 (0.65 – 3.15) |  | 1.15 (0.48 – 2.76) |  |
| High chicken management score | 0.87 (0.54 – 1.43) |  | 0.97 (0.59 – 1.60) |  | 1.11 (0.43 – 2.83) |  | 1.00 (0.28 – 3.60) |  |
| **Diarrhea by sex** |  | 0.141 |  | 0.767 |  | 0.327 |  | 0.383 |
| Boys | 0.57 (0.26 – 1.24) |  |  |  | 1.56 (0.72 – 3.40) |  | 1.35 (0.62 – 2.92) |  |
| Girls | 1.20 (0.66 – 2.20) |  |  |  | 0.98 (0.45 – 2.16) |  | 0.83 (0.32 – 2.14) |  |

^1^Values are RR (95% CI) in columns 2, 4, 6, and 8. The p-value for effect modification is reported in columns 3, 5, 7, and 9. Robust bootstrapped confidence intervals are clustered at the village level and reported in exponentiated form. Models adjusted for the baseline value of the outcome. Anemia data were not collected at midline. Child anemia was defined as a hemoglobin value less than 11 g/dl, after adjusting for altitude. Low or high chicken management score was a binary variable defined as being above or below the median chicken management score at baseline. ACGG=African Chicken Genetic Gains; ATONU=Agriculture to Nutrition; CI=Confidence Interval; RR=Risk Ratio.
